# Yield Performance, Resource-Use Efficiency, and Economic Profitability from Adopting Soybean-Based Cotton/Maize/Sugarcane Intercropping Systems Under Arid-Irrigated Conditions

**DOI:** 10.3390/plants15142111

**Published:** 2026-07-08

**Authors:** Hassan Shehryar Yasin, Muhammad Ali Raza, Lingyang Feng, Jiqin Han

**Affiliations:** 1 The College of Economics and Management, Nanjing Agricultural University, Nanjing 210095, China; 2 National Research Center of Intercropping, The Islamia University of Bahawalpur, Bahawalpur 63100, Pakistan; 3 Institute of Advanced Agricultural Sciences, Peking University, Weifang 261325, China

**Keywords:** diversification, farming systems, sustainability, complementarity, agroecology, synchronization

## Abstract

Legume intercropping is a productive diversification strategy that can improve land-use efficiency and farm profitability, particularly for smallholders. However, its adoption remains limited in resource-intensive farming systems because crop-specific agronomic performance, input-use implications, and economic feasibility are not well documented under farmer-field conditions. This four-year field study (2021–2024) evaluated four sole cropping systems (sole cotton, sole maize, sole sugarcane, and sole soybean) and three additive soybean-based intercropping systems (cotton/soybean, maize/soybean, and sugarcane/soybean) under arid-irrigated conditions. Crop yield, dry matter accumulation, nutrient uptake, land equivalent ratio for land (LER_L_), land equivalent ratio for nitrogen (LER_N_), land equivalent ratio for phosphorus (LER_P_), economic profitability, and labor requirement were assessed. On average, across the four study years, intercropped cotton, maize, and sugarcane produced 80%, 74%, and 88% of their respective sole-crop yields, while intercropped soybean produced 72%, 59%, and 83% of sole-soybean yield in cotton/soybean, maize/soybean, and sugarcane/soybean intercropping systems, respectively. At the system level, the total LER_L_, LER_N_, and LER_P_ values ranged from 1.33 to 1.71, 1.35–1.68, and 1.25–1.64, respectively, indicating resource-use (land and nutrients) advantages of intercropping compared with sole cropping. Based on these observed LER_N_ and LER_P_ values, soybean-based intercropping showed theoretical potential to reduce nitrogen and phosphorus fertilizer requirements by 26–40% and 20–39%, respectively; however, these estimates should be interpreted as potential input-economy indicators rather than experimentally validated fertilizer reductions. Economically, intercropping increased net income by ≈29–154% and generated 18–28% more labor demand than the corresponding sole systems, with sugarcane/soybean showing the highest net income (2937 USD ha^−1^). Overall, additive soybean-based intercropping, particularly cotton/soybean and sugarcane/soybean systems with greater temporal niche differentiation, improved land productivity, nutrient-use efficiency indicators, and farm profitability under the tested arid-irrigated conditions. Further multi-location studies with actual reduced-fertilizer treatments are needed to validate fertilizer-saving potential and broader applicability.

## 1. Introduction

Sustainably enhancing crop production while lowering environmental pollution is a complex problem that demands a collective approach from agricultural scientists, policymakers, and industry stakeholders [1,2]. Since 1960, crop production has doubled, driven by genetic improvements, increased pesticide and fertilizer use, and improved water and nutrient management practices [3,4]. Still, crop yields need to be increased by at least 50% to maintain food security and economic sustainability for an estimated global human population of approximately nine billion [5,6]. Therefore, farmers are using water and chemical fertilizers at rates higher than the crops actually require [3,7], primarily in sole-cropping systems (SCs)—cropping practices where a single crop species is grown on a field at one time—a dominant method for conventionally grown major crops, e.g., cotton, maize, sugarcane, and wheat, in the world [8]. Moreover, current technology development programs, farm machinery, marketing, infrastructure, and subsidies continue to promote SCs, as they are easy to adopt, practical to implement, and produce high crop yields—a key prerequisite for adopting any agricultural practice [8,9]. Nevertheless, despite their significant contributions to global crop production, SCs face criticism for their documented detrimental impacts on soil health, water quality, and climate change [10]. Consequently, researchers are communicating that these systems threaten the future sustainability of farming activities and the delivery of food system outcomes, including a stable and healthy food supply, preserved biodiversity, and farm incomes [4,11]. Thus, these challenges necessitate the development of innovative crop production methods for sustainable and resilient agriculture [4,10,11,12,13,14].

Intercropping, a cropping strategy for growing two or more crops simultaneously, has recently been termed the new green revolution due to its potential to increase crop yields and monetary benefits with reduced inputs compared to SCs [6,12]. Specifically, legume-based intercropping systems (LBIs) are the most common intercropping systems (ICs); these systems are developed and optimized in accordance with ecological principles to enhance resource-use efficiency and offer ecological services by reducing the use of chemical inputs (pesticides and fertilizers) and mitigating their environmental impact [11,15,16,17]. Consequently, these systems are increasingly gaining attention in developing and developed countries, primarily for enhancing the resilience and sustainability of current food systems [9,18]. Recent studies have revealed that adopting LBIs can enhance crop diversity and promote the complementary use of available (land and light) and applied (water and chemical nutrients) resources [6,19,20,21]. Previous research has provided increasing evidence that intercropping can outperform monocropping by improving land productivity, resource capture, and economic returns. For example, cereal-legume and cash crop-legume intercropping systems have been reported to increase total system productivity and land equivalent ratio through complementary canopy architecture, asynchronous growth duration, and improved use of light, water, and nutrients [4,20,22]. Legume-based systems can also improve nitrogen use at the system level because the legume component can acquire part of its nitrogen through biological fixation, thereby reducing direct competition with the companion non-legume crop for soil nitrogen [9,23]. In addition, previous studies on maize/soybean, cotton/soybean, and sugarcane/soybean intercropping have shown that suitable crop combinations and row configurations can improve yield stability, increase net economic returns, and enhance the practical feasibility of diversified cropping systems compared with monocropping [14,24,25]. These findings indicate that the benefits of intercropping depend strongly on crop combination, planting configuration, growth-stage synchronization, and local management conditions. However, compared to global trends, the adoption of LBIs in the sub-continent—home to one-third of the world population—remains slow. For instance, LBIs cover more than 15% of the global arable land [2,26], whereas in the sub-continent, less than 1% of the arable area is under intercropping. Specifically, in Pakistan, the main focus of this study, intercropping adoption is negligible, mainly due to a lack of training and extension services, as well as concerns regarding profitability, practicality, and the availability of suitable farm machinery [27]. Therefore, it is important to develop area- and crop-specific ICs that can improve farm productivity and resource-use efficiency, especially under the current scenario of increasing climate extremes and recurrent drought incidence [28], as reported by several studies [4,12,17].

Increasing land productivity is essential worldwide to meet the growing food demand without converting additional land for agricultural production [18]. In this context, Pakistan faces huge pressure to achieve higher land productivity and ensure national food security, particularly for grain, sugar, and fiber crops, given that it has only 0.13 hectares of arable land per capita. In addition, as in other countries, the Pakistani government is also encouraging farmers to reduce fertilizer use to mitigate its adverse environmental effects and address issues such as soil acidification and farm eutrophication [16,27]. Hence, in the current scenario of climate change and unpredictable limited resources, LBIs present an opportunity to improve crop yields and achieve higher economic returns while increasing resource-use efficiency [22,29]. The inclusion of legumes in cropping systems promotes diversity and may help break disease and pest cycles that often arise from growing a single crop repeatedly in one region [10,30] and may improve nitrogen and phosphorus availability through biological nitrogen fixation and root-mediated nutrient mobilization [9,29,31]. Despite being widely recognized for its agronomic and economic benefits, intercropping lacks comprehensive scientific data, especially from a farmer’s field under arid-irrigated conditions, on its impacts on crop yield variations and economic returns, predominantly regarding the outcomes of intercropping an additional legume (soybean) with main crops such as cotton, maize, and sugarcane within ICs. Although adoption constraints are discussed here to contextualize the limited use of legume-based intercropping in Pakistan, the present study did not directly investigate farmer adoption behavior or socio-economic decision-making. Instead, it evaluates the agronomic performance, nutrient uptake, land-use efficiency, and economic feasibility of soybean-based intercropping systems under farmer-field conditions, which are important prerequisites for future adoption-oriented research. Therefore, this field study was conducted for four consecutive years on a progressive farmer’s field with the objectives of (i) quantifying the effects of soybean-based intercropping systems (SBIs: cotton/soybean, maize/soybean, and sugarcane/soybean) on dry matter, nutrient uptake, and crop yield, (ii) evaluating land-use and nutrient-uptake efficiency using LER-based indicators, and (iii) assessing economic profitability and labor requirement under arid-irrigated conditions. Thus, we hypothesized that adopting SBIs could improve crop productivity, land-use efficiency, nutrient-uptake efficiency, and economic returns compared with the corresponding sole cropping systems, while enabling farmers to produce an additional protein-rich legume crop without reducing the main crop population. By focusing on crop yield, dry matter accumulation, nutrient uptake, LER-based indicators, and crop-combination performance, this study contributes to applied crop production and intercropping research, while the economic indicators are used to evaluate the practical feasibility of these plant production systems under farmer-field conditions.

## 2. Materials and Methods

### 2.1. Site Description

**Figure 1:**
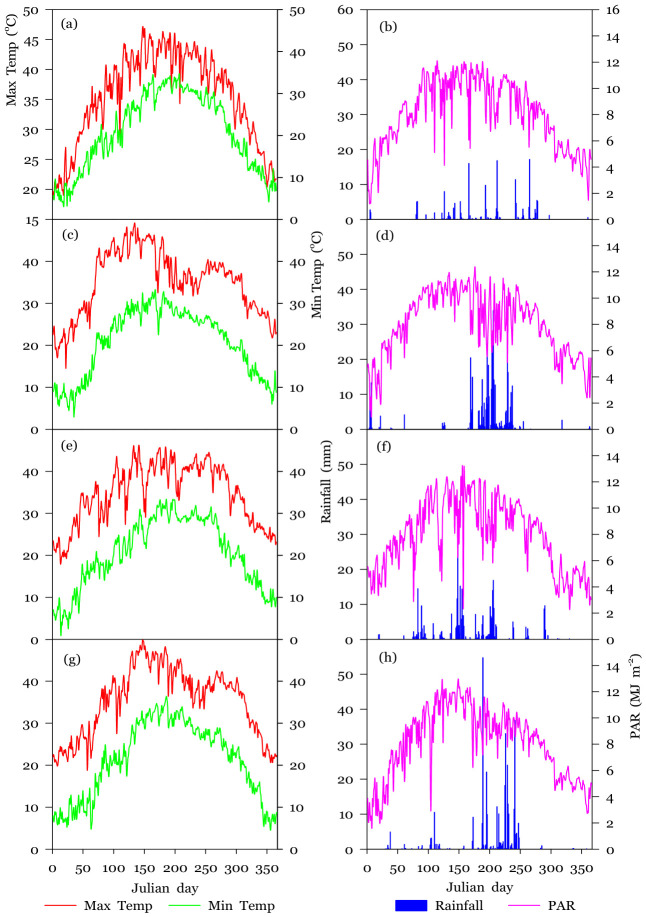
Daily maximum temperature (Max Temp), minimum temperature (Min Temp), rainfall, and photosynthetically active radiation (PAR) at the study site during the growth period of different crops in 2021 (**a**,**b**), 2022 (**c**,**d**), 2023 (**e**,**f**), and 2024 (**g**,**h**).

Punjab is the primary region where farmers practice SBIs, currently accounting for 85–90% of the total area under this practice in Pakistan. A representative farmer’s field, where SBIs have been practiced since 2018, was selected in Khairpur Tamewali (29.57° N, 72.15° E; altitude 130 m). Field experiments were conducted for four consecutive years (2021–2024) at MARS-FARMS under the supervision of a team of scientists from the National Research Center for Intercropping (NRCI), located at the Islamia University of Bahawalpur, District Bahawalpur, Southern Punjab, Pakistan. The research site has a continental monsoon climate, with a mean temperature of 25.7 °C and an average annual rainfall of 143 mm. The experimental site’s soil has a sandy loam texture with a pH of 7.8, organic matter of 5.3 g kg^−1^, total nitrogen (N) of 0.5 g kg^−1^, total phosphorus (P) of 5.7 mg kg^−1^, potassium (K) of 85.4 mg kg^−1^, and bulk density of 1.5 Mg m^−3^. The details of the weather conditions of the experimental site are presented in Figure 1. Generally, local farmers in this region predominantly practice resource-intensive SCs for maize, sugarcane, and cotton production, as they are well-established major crops known for higher economic returns in the arid-irrigated regions. Thus, we selected cotton, maize, and sugarcane as the main crops, and soybean was selected as the legume intercrop because, since 2021, the Punjab government has been encouraging farmers to intercrop legumes, mainly soybean, to increase farm income and enhance soil fertility [24].

### 2.2. Trial Management

**Figure 2:**
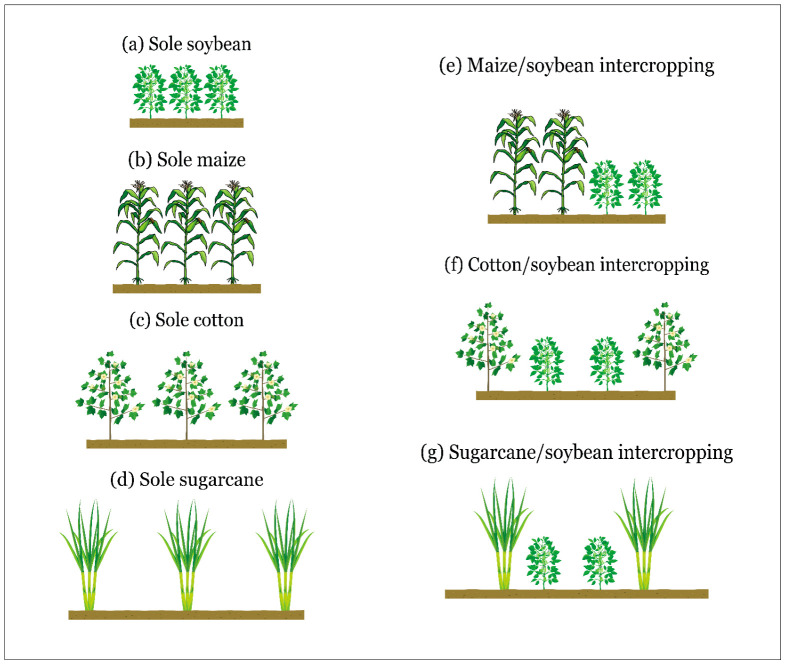
Field layout for cultivating main crops (maize, cotton, and sugarcane) and soybean under sole and soybean-based intercropping systems. In cotton/soybean and sugarcane/soybean intercropping, two soybean rows were intercropped between every two rows of cotton or sugarcane, while in the maize/soybean intercropping, two maize rows were alternated with two rows of soybean in strips.

The experiment was conducted for four consecutive years (from 2021 to 2024) in the same farmer’s field within an approximately 4-ha area. A randomized complete block design was used each year, with three blocks as replications. Each block contained all seven treatments: four sole cropping systems (sole cotton, sole maize, sole sugarcane, and sole soybean; Figure 2a–d) and three soybean-based intercropping systems (cotton/soybean, maize/soybean, and sugarcane/soybean; Figure 2e–g). Thus, each year consisted of 21 experimental plots. Treatments were randomly assigned to plots within each block every year, and plots were re-randomized annually within the same experimental area. Therefore, the year effect represents environmental variation among years rather than repeated measurements from the same fixed plots. Each experimental plot measured 1500 m^2^ (50 m × 30 m). The total area occupied by the seven plots within each block was 10,500 m^2^ (1500 m^2^ × 7), excluding buffer spaces. A 2 m buffer zone was maintained between adjacent plots and between blocks to reduce interference among treatments. Crop rows were oriented north–south throughout the experiment. To minimize border effects, crop measurements and yield harvesting were conducted from the central area of each plot, excluding border rows and end plants. The intercropping treatments followed an additive intercropping design, in which the full recommended plant density of the main crop was maintained and soybean was introduced as an additional crop in the inter-row space. Consequently, total plant density per unit area was higher in the intercropping systems than in the corresponding sole main-crop systems. This design reflects the locally practiced farmer-field configuration in the study region and was selected to evaluate the practical performance of soybean addition without reducing the main crop population. For cotton/soybean and sugarcane/soybean intercropping, two soybean rows were grown between cotton or sugarcane rows, whereas for maize/soybean intercropping, two soybean rows were grown with two maize rows (Figure 2e–g). The same planting configurations were maintained in all four years. Cotton, maize, and soybean were grown at 50,000, 90,000, and 167,000 plants ha^−1^, respectively; sugarcane was planted at 57,000 two-bud cane setts ha^−1^ in both sole and intercropping systems [6,24,32]. Detailed planting configurations, including row spacing, row ratios, strip distance, plant spacing, and plant density, are provided in Supplementary Table S1.

**Figure 3:**
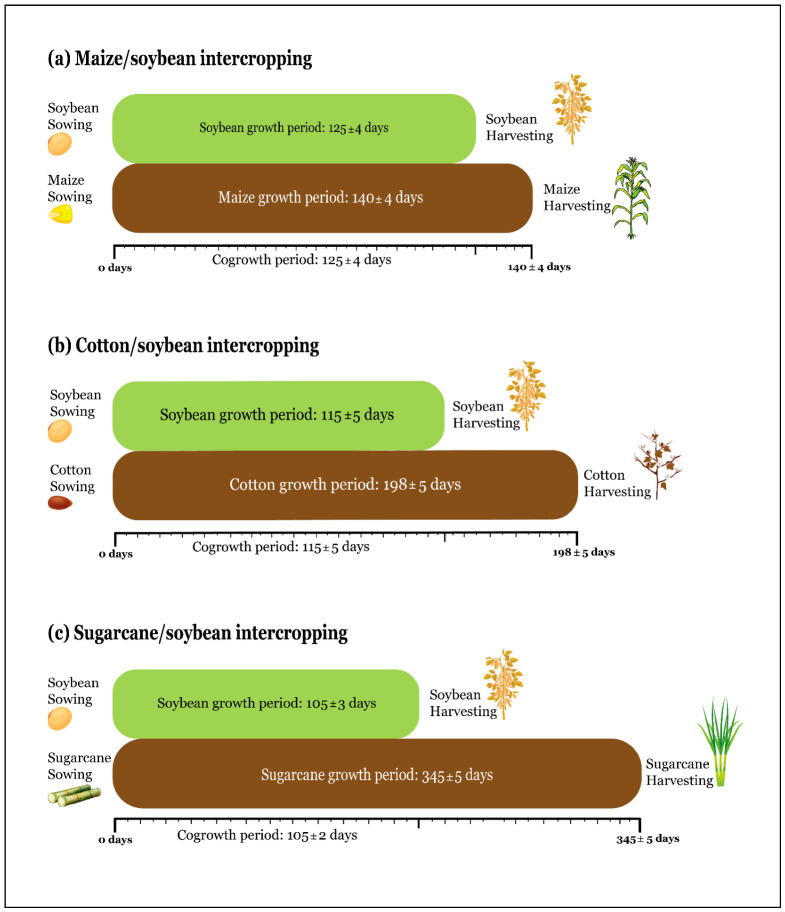
Crop growth period of main crops (maize, cotton, and sugarcane) and soybean under soybean-based intercropping systems. The upper green bar represents the growth period of soybean, while the lower dark-brown bar shows the growth period of the main crops in soybean-based intercropping systems. The cogrowth period indicates the number of days when the main crops and soybean grow together in soybean-based intercropping systems. Each bar reflects the average growth days for each crop, based on four years of experimentation across different cropping systems. Notably, (**a**) in maize/soybean intercropping, soybean’s vegetative growth period was 53 ± 3 days and reproductive growth period was 72 ± 1 days, while maize’s vegetative growth period was 63 ± 1 days and reproductive growth period was 77 ± 3 days; (**b**) in cotton/soybean intercropping, soybean’s vegetative growth period was 50 ± 2 days and reproductive growth period was 65 ± 3 days, while cotton’s vegetative growth period was 70 ± 4 days and reproductive growth period was 128 ± 1 days; and (**c**) in sugarcane/soybean intercropping, soybean’s vegetative growth period was 42 ± 2 days and reproductive growth period was 63 ± 1 days, while sugarcane’s initial growth period was 120 ± 1 days, grand growth phase was 150 ± 3 days, and maturation growth period was 75 ± 1 days. The growth periods and growth stage durations of sole maize, sole cotton, and sole sugarcane were identical to those of intercropped maize, cotton, and sugarcane, while the growth period and growth stage durations of sole soybean were the same as those of soybean intercropped with sugarcane.

In all years of this study, cotton variety ‘IUB-13’, maize hybrid ‘DK-7024’, sugarcane variety ‘CPF-237’, and the soybean line ‘CAS-SOY-171’ were planted in the first week of February. In contrast to the local practice, where farmers typically grow and maintain sugarcane for two to three years as a ratoon crop, this study did not use ratoon sugarcane in any of the tested years, as the farmer prefers to plant fresh sugarcane every year. Each year, across all sole and intercropping treatments, maize was harvested during the last week of June, whereas sugarcane was harvested during the second week of January. Cotton was picked three times, during the third week of June, the last week of July, and the second week of August. Sole soybean and soybean intercropped with sugarcane were harvested during the third week of May, whereas soybean intercropped with cotton and maize was harvested during the last week of May and the second week of June, respectively. The harvesting times of soybean differed among intercropping systems due to variations in crop microenvironment, shading effects, and maturity synchronization with the companion crops under different intercropping conditions. Crop growth periods of cotton, maize, sugarcane, and soybean in ICs and SCs are shown in Figure 3. For sole and intercropped cotton, P at 85 kg ha^−1^ was applied as a basal dose, and N at 300 kg ha^−1^ was used in five split doses (each of 60 kg ha^−1^) at 30 ± 02, 55 ± 05, 80 ± 05, 105 ± 05, and 130 ± 05 days after cotton germination. For sole and intercropped maize, P at 85 kg ha^−1^ was applied as a basal dose, and N at 300 kg ha^−1^ was used in five split doses (each of 60 kg ha^−1^) at 25 ± 03, 35 ± 03, 45 ± 03, 55 ± 05, and 65 ± 05 days after maize germination; For sole and intercropped sugarcane, P at 120 kg ha^−1^ was applied in three split doses (each of 40 kg ha^−1^): the first as a basal dose, and the second and third doses at 90 ± 05 and 130 ± 05 days after sugarcane sprouting, and N at 300 kg ha^−1^ was used in five split doses (each of 60 kg ha^−1^) at 45 ± 05, 90 ± 05, 135 ± 05, 165 ± 05, and 195 ± 05 days after sugarcane sprouting. Whereas, for sole soybean, P at 85 kg ha^−1^ was applied as a basal dose, and the N at 80 kg ha^−1^ was applied in two split doses (each of 40 kg ha^−1^) at 30 ± 1 and 50 ± 5 days after soybean germination. For irrigation, we used the furrow-irrigation method, with a water supply of 600 ± 100 mm, 550 ± 100 mm, 1800 ± 100 mm, and 450 ± 50 mm for sole cotton, maize, sugarcane, and soybean, respectively. Notably, in all years, the intercropped soybean was always obtained using the same fertilizer and irrigation as supplied to sole cotton, maize, and sugarcane, i.e., without any additional or dedicated fertilizer or irrigation application.

### 2.3. Crop Measurements, Nutrient Uptake, and Resource-Use Efficiency

Dry matter accumulation and N and P uptake of cotton, maize, sugarcane, and soybean were measured to determine the growth differences in all crops in SCs and ICs. For this purpose, at the maturity of each crop, an area of 1 m^2^ was manually harvested from each cropping system and replication. Then, the harvested samples were dried and processed, and the dry matter and nutrient uptake were determined following the methods described in our previous studies, with dry matter expressed in tons (t) ha^−1^ and nutrient uptake expressed in kg ha^−1^ [16,23]. Crop yield was measured separately from each plot in every year. For maize, soybean, and sugarcane, a central harvest area of 900 m^2^ (30 m × 30 m) was marked within each 1500 m^2^ plot, after excluding border rows. Maize grain and soybean seed yields were determined after sun drying for 10–25 days and machine threshing, and were expressed as t ha^−1^. Sugarcane yield was determined from the same central harvest area by harvesting and weighing cane yield, which was then expressed as t ha^−1^. For cotton, a central area of 400 m^2^ (20 m × 20 m) was marked within each plot for seed cotton yield measurement. Seed cotton was hand-picked three times from this area, and the cumulative yield from all pickings was calculated and expressed as t ha^−1^.

To evaluate the land use advantage of ICs compared to SCs, partial land equivalent ratios were calculated for the main crops (cotton, maize, or sugarcane; pLER_M_) and soybean (pLER_S_) as per the following equations [18,19]:

(1) $$p{LER}_{M}=\frac{{GY}_{IM}}{{GY}_{SM}}$$

(2) $$p{LER}_{S}=\frac{{GY}_{IS}}{{GY}_{SS}}$$

Here, the GY_IM_ and GY_IS_ are the crop yield of main and soybean crops in ICs, and the GY_SM_ and GY_SS_ are the crop yield of main and soybean crops in SCs. After calculating the pLER_M_ and pLERs, the total land use advantage (Total LER_L_) for each IC was estimated as per the following equation [18,19]:

(3) $$Total\;{LER}_{L}=p{LER}_{M}+p{LER}_{S}$$

Potential input economy for N and P was estimated using the analogy of LER for N and P. For this purpose, we first calculated the partial land equivalent ratios for N and P of each crop as per the following equations [33]:

(4) $$p{LER}_{NM}=\frac{{NU}_{IM}}{{NU}_{SM}}$$

(5) $$p{LER}_{NS}=\frac{{NU}_{IS}}{{NU}_{SS}}$$

(6) $$p{LER}_{PM}=\frac{{PU}_{IM}}{{PU}_{SM}}$$

(7) $$p{LER}_{PS}=\frac{{PU}_{IS}}{{PU}_{SS}}$$

Here, the pLER_NM_ and pLER_PM_ are the partial land equivalent ratios for N and P of the main crops, and pLER_NS_ and pLER_PS_ are the partial land equivalent ratios for N and P of the soybean, respectively. The NU_IM_ and NU_IS_ are the N uptake of main and soybean crops in ICs, NU_SM_ and NU_SS_ are the N uptake of main and soybean crops in SCs, PU_IM_ and PU_IS_ are the P uptake of main and soybean crops in ICs, and PU_SM_ and PU_SS_ are the P uptake of main and soybean crops in SCs, respectively. Afterwards, the total LER for N (LER_N_) and P (LER_P_) were estimated as per the following equations [33]:

(8) $$Total\;{LER}_{N}=p{LER}_{NM}+p{LER}_{NS}$$

(9) $$Total\;{LER}_{P}=p{LER}_{PM}+p{LER}_{PS}$$

Total LER value for N or P greater than one indicates the benefits of intercropping, suggesting that ICs are more productive and efficient in utilizing the available N or P fertilizers than their SCs. Based on this data, we further calculated the potential savings in fertilizer applications and the associated capital investment by comparing the fertilizer requirements of ICs and SCs. This allowed us to quantify the potential reductions in fertilizer use and associated costs in ICs relative to SCs. Thus, we calculated the estimated N or P fertilizer requirement for ICs as per the following equations:

(10) $${Fertilizer}_{N}\;required\;in\;ICs=\frac{{Fertilizer}_{N}\;applied\;to\;SCs}{Total\;{LER}_{N}}$$

(11) $${Fertilizer}_{P}\;required\;in\;ICs=\frac{{Fertilizer}_{P}\;applied\;to\;SCs}{Total\;{LER}_{P}}$$

These equations are logically derived from the established interpretation of LER_N_ and LER_P_ as measures of nutrient use efficiency [33]. If intercropping systems are LER_N_ times more efficient in nitrogen uptake than sole crops, then theoretically, they could achieve the same total uptake with proportionally less fertilizer, leading to the inverse relationship used above. Subsequently, we estimated the potential N or P fertilizer savings/potential N or P reductions in ICs compared to SCs using the following equations:

(12) $${Fertilizer\;saving}_{N}={Fertilizer}_{N}\;applied\;to\;SCs-\;{Fertilizer}_{N}\;required\;in\;ICs$$

(13) $${Fertilizer\;saving}_{P}={Fertilizer}_{P}\;applied\;to\;SCs-{Fertilizer}_{P}\;required\;in\;ICs$$

A higher estimated N or P fertilizer-saving value indicates greater theoretical input-economy potential under the assumption that crop yield could be maintained with proportionally reduced fertilizer inputs. Finally, the total cost savings on fertilizer inputs in each intercropping system were calculated using the following equations:

(14) $${Cost\;{saving}_{N}=Fertilizer}_{N}\;saving\;\times Cost\;per\;unit\;of\;{fertilizer}_{N}$$

(15) $${Cost\;{saving}_{P}=Fertilizer}_{P}\;saving\times Cost\;per\;unit\;of\;{fertilizer}_{P}$$

(16) $$Total\;cost\;saving={Cost\;saving}_{N}+{Cost\;saving}_{P}$$

A detailed step-by-step worked example demonstrating these calculations for the sugarcane/soybean intercropping system is provided in Supplementary Table S2. Importantly, the calculations of fertilizer requirements, savings, and cost savings presented above represent theoretical estimates of reduction potential based on the observed nutrient use efficiency (LER_N_ and LER_P_) in intercropping compared to sole cropping systems. These estimates assume that fertilizer inputs could be reduced in proportion to the efficiency gains without compromising crop yield. However, the experiment did not include treatments with reduced fertilizer application rates; all intercropping systems in this study received the same fertilizer inputs as their respective sole crops (as described in Section 2.2). Therefore, these estimates should be interpreted as indicators of efficiency gains and potential input economy, not as experimentally validated reductions. This approach follows established methodology in intercropping research for assessing nutrient use efficiency through LER for N and P [33]. The assumptions underlying these theoretical estimates are provided in Supplementary Table S3.

### 2.4. Economic Analysis

Economic performance of each cropping system was assessed using standard budgeting methods to estimate the total production cost, gross income, net income, benefit-to-cost ratio (BCR), and labor requirement. Production costs were calculated separately for each year using year-specific local market prices and actual field-operation records. The cost components included crop seed or cane sets, land preparation, urea fertilizer, single super phosphate fertilizer, pesticides/weedicides/fungicides, irrigation, and labor costs for field operations such as sowing, fertilizer application, irrigation, weeding, pesticide or weedicide spraying, harvesting, threshing, and cotton picking. Input prices, crop sale prices, and labor wage rates were obtained from local market records and farmer-field payment records for each experimental year. All costs and returns were first recorded in Pakistani Rupees (PKR) and then converted into US dollars (USD) using the average annual exchange rate for each year. Labor use was expressed as labor requirement in person-days ha^−1^ rather than as the number of unique individual workers. For each cropping system, hired labor was recorded daily throughout the growing season for each field operation. Total labor cost was calculated by multiplying the number of person-days required for each operation by the corresponding daily wage rate in that year. Thus, higher person-days ha^−1^ indicate greater labor demand and employment-generation potential, but they also represent an additional input cost and management requirement for farmers. The detailed year-wise costs of inputs, field operations, labor costs, working days, and labor requirement are provided in Supplementary Tables S4 and S5. After that, the net income and benefit-to-cost ratio (BCR) were calculated using the following equations:

(17) Net income=Gross income−Total cost

(18) $$BCR=\frac{Gross\;income}{Total\;production\;cost}$$

Gross income was calculated by multiplying crop yield by the corresponding year-specific local market price of each crop. The market prices of cotton, maize, sugarcane, and soybean were recorded separately for each year at harvest time. The average exchange rates used for conversion were 1 USD = PKR 163 in 2021, PKR 204 in 2022, PKR 290 in 2023, and PKR 277 in 2024.

In addition, to assess the potential implications of fertilizer reduction, a qualitative sensitivity analysis was conducted (Supplementary Table S6), evaluating yield risk under three reduction scenarios (10%, 20%, and 30%) based on observed LER values for N and P and temporal niche differentiation of each intercropping system. To complement the mass-based and land-use efficiency metrics, we calculated two value-based indices for each intercropping system: (i) system gross value (SGV), the total market value of all crops harvested from one hectare, calculated as SGV = (Yield_main crop_ × Price_main crop_) + (Yield_soybean_ × Price_soybean_), and (ii) monetary advantage index (MAI), the economic advantage of intercropping compared to sole cropping, calculated as MAI = (Value of both intercrops) × (LER − 1)/LER, here value of both intercrops is the SGV of the intercropping system, and LER is the total land equivalent ratio for that system [34].

### 2.5. Statistical Analysis

Data were analyzed using a generalized linear mixed effect model (GLMM) in SPSS 29 (IBM Corp., Armonk, NY, USA). The experiment was laid out in a randomized complete block design with three replications per treatment. The following model was used for all response variables:

$$Y_{ijk}=\mu \;+\alpha_{i}+\beta_{j}+{\left(\alpha \beta \right)}_{ij}+\gamma_{k}+\varepsilon_{ijk}$$

where Y_ijk_ is the response variable, μ is the overall mean, α*_i_* is the fixed effect of cropping system (i = sole cotton, sole maize, sole sugarcane, sole soybean, cotton/soybean, maize/soybean, sugarcane/soybean), β_j_ is the fixed effect of year (j = 2021, 2022, 2023, 2024), (αβ)_ij_ is the interaction between cropping system and year, γ_k_ is the random effect of block (k = 1, 2, 3), and ε_ijk_ is the residual error. Both γ_k_ and ε_ijk_ were assumed to be normally distributed with constant variances. For total system yield and total system nutrient uptake, which compare only the three intercropping systems, the same model was used with cropping system (i) representing only cotton/soybean, maize/soybean, and sugarcane/soybean. Complete model details for each response variable are provided in Supplementary Table S7, which uses software-style notation for clarity. This analysis enabled us to evaluate the effects of years and cropping systems on (i) growth indicators (dry matter and nutrient uptake) and yield performance, and (ii) resource utilization (estimated as the LER for land, N, and P) and potential resource reduction associated with the adoption of each intercropping system separately compared to their SCs (mixed model ANOVAs are provided in Supplementary Tables S8–S15). The statistical analysis accounted for the combined effect of years, cropping systems, and their interaction. The years and cropping systems were treated as fixed factors in the GLMM analysis, while replications were considered random effects. Data in tables and figures are shown as means with ± standard errors. The significant differences between ICs and SCs were assessed using the LSD test, with a significance level set at *p* < 0.05.

## 3. Results

### 3.1. Growth Indicators

Crop productivity, measured as the dry matter and nutrient uptake, was significantly influenced by cropping systems; the impact of years on crop productivity parameters of main crops (cotton, maize, and sugarcane) and intercropped soybean varied, with significance differing across dry matter and nutrient uptake; and their interactions were always non-significant for all parameters, except for soybean in maize/soybean intercropping (Supplementary Tables S8–S11). Overall, all main crops (cotton, maize, and sugarcane; Table 1) and soybean (Table 2) had higher dry matter and nutrient uptake in SCs than in ICs. However, on average, across the four years, sugarcane recorded the highest dry matter, N uptake, and P uptake in sugarcane/soybean intercropping, followed by cotton in cotton/soybean intercropping, and maize in maize/soybean intercropping. Soybean intercropped with sugarcane achieved higher dry matter, N uptake, and P uptake than when intercropped with cotton and maize, suggesting that differences in crop growth duration may have influenced growth performance and nutrient uptake of the component crops in ICs, thereby influencing growth indicators in ICs. Moreover, over the years, on average, the total system N or P uptake (main crop N/P uptake + soybean N/P uptake) of all intercropping systems was significantly higher than their respective values in SCs (Table 3). Compared with their corresponding sole main-cropping systems, the total system N and P uptake were 43% and 24% higher in cotton/soybean intercropping, 19% and 9% higher in maize/soybean intercropping, and 47% and 35% higher in sugarcane/soybean intercropping, respectively. Collectively, these results indicate that soybean-based intercropping increased system-level nutrient uptake compared with the corresponding sole main-cropping systems, particularly in cotton/soybean and sugarcane/soybean intercropping.

### 3.2. Yield Performance

Cropping systems significantly affected the crop yield and total system yield (main crop yield + soybean crop yield), while years showed a non-significant effect for maize, sugarcane, intercropped soybean yield, and total system yield, except for cotton yield; and their interaction consistently had a non-significant effect on crop yields and total system yield (Supplementary Tables S11 and S12). In all years, sugarcane always achieved the highest yield, followed by maize, soybean, and cotton, and across all cropping systems, crop yields were higher in SCs than in ICs (cotton, maize, and sugarcane, Table 1; and soybean, Table 2). On average, cotton, maize, and sugarcane achieved 80%, 74%, and 88% of sole cotton, maize, and sugarcane yields in ICs, respectively. Whereas soybean intercropped with cotton, maize, and sugarcane achieved 72%, 59%, and 83% of sole soybean yield in ICs, respectively. When averaged across the four years, the total system yield of maize/soybean (maize yield + soybean yield) and sugarcane/soybean (cane yield + soybean yield) intercropping was 13% and 10% lower than the sole maize and sugarcane yields, respectively (Table 3). However, the total system yield of cotton/soybean (cotton yield + soybean yield) was 80% higher than the sole cotton yield (Table 3). Overall, crop growth productivity and yield trends remained consistent over the four years, demonstrating the potential of SBIs to increase legume production without requiring additional sole-soybean land, while maintaining or slightly reducing the yield of the main crop.

### 3.3. Intercropping Advantage

**Figure 4:**
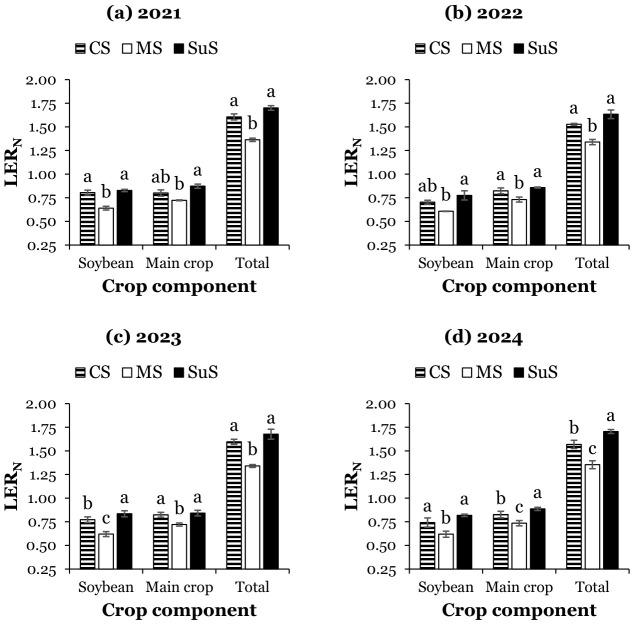
Partial and total land equivalent ratio for nitrogen (LER_N_) of soybean-based intercropping systems. Soybean and main-crop values represent partial land equivalent ratio for nitrogen (pLER_N_), whereas total values represent the sum of both component values. Different lowercase letters indicate significant differences among intercropping systems within the same crop component at *p* ≤ 0.05. CS, cotton/soybean; MS, maize/soybean; SuS, sugarcane/soybean intercropping systems.

**Figure 5:**
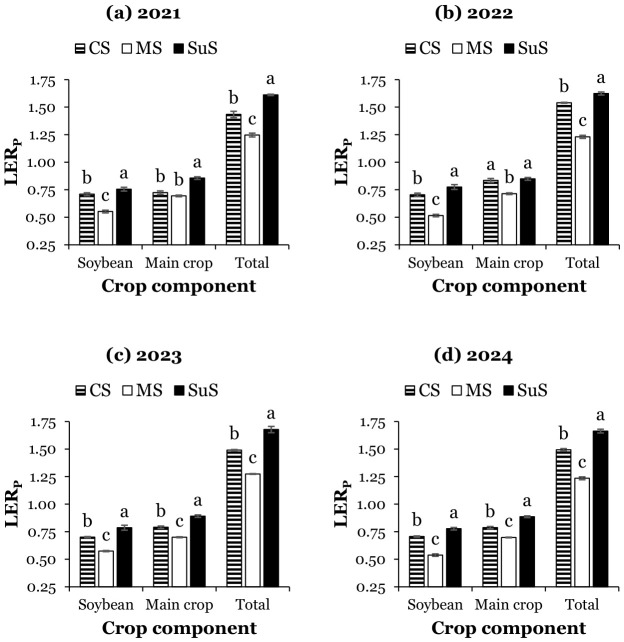
Partial and total land equivalent ratio for phosphorus (LER_P_) of soybean-based intercropping systems. Soybean and main-crop values represent partial land equivalent ratio for nitrogen (pLER_P_), whereas total values represent the sum of both component values. Different lowercase letters indicate significant differences among intercropping systems within the same crop component at *p* ≤ 0.05. CS, cotton/soybean; MS, maize/soybean; SuS, sugarcane/soybean intercropping systems.

**Figure 6:**
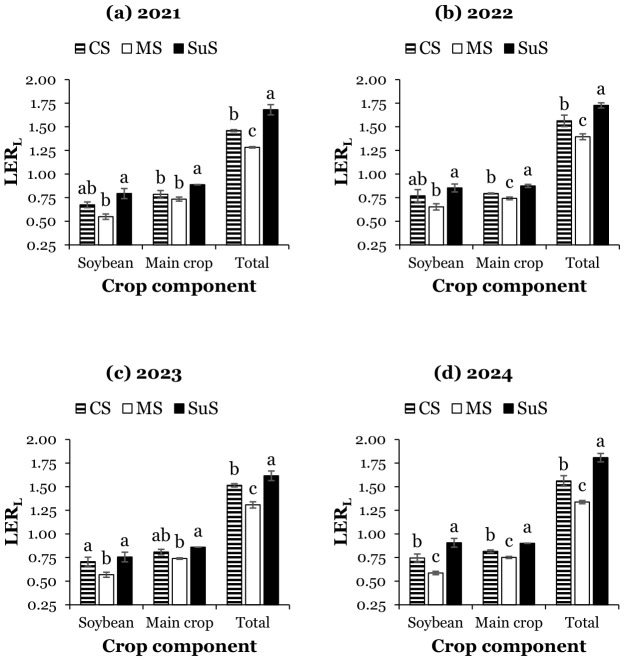
Partial and total land equivalent ratio for land (LER_L_) of soybean-based intercropping systems. Soybean and main-crop values represent partial land equivalent ratio for nitrogen (pLER_P_), whereas total values represent the sum of both component values. Different lowercase letters indicate significant differences among intercropping systems within the same crop component at *p* ≤ 0.05. CS, cotton/soybean; MS, maize/soybean; SuS, sugarcane/soybean intercropping systems.

Years showed non-significant effects on all crop’s partial and total LER values for N (LER_N_), P (LER_P_), and land (LER_L_), except for pLER value for P of main crops; however, cropping systems significantly impacted the partial and total LER values for N, P, and land of main crops and soybean, and the interactions between cropping systems × years for the values of partial and total LER for N, P, and land were also non-significant for all crops, except the interactions for the values pLER_P_ of main crops (Supplementary Table S13). Generally, across all ICs and years, the total LER values for land, N, and P were always greater than one, indicating intercropping advantage over SCs (LER_N_: Figure 4; LER_P_: Figure 5; and LER_L_: Figure 6). Specifically, when averaged over the four years, soybean intercropped with sugarcane had the highest pLER_L_, pLER_N_, and pLER_P_ values, while soybean intercropped with maize had the lowest pLER_L_, pLER_N_, and pLER_P_ values. Whereas among the main crops, sugarcane in sugarcane/soybean intercropping consistently achieved higher pLER_L_, pLER_N_, and pLER_P_ values than cotton in cotton/soybean intercropping and maize in maize/soybean intercropping (Figure 4, Figure 5 and Figure 6). On average, over the four years, among the ICs, sugarcane/soybean intercropping achieved the highest total LER_L_ (1.71), LER_N_ (1.68), and LER_P_ (1.64) values. These values were 12%, 7%, and 10% higher than those of cotton/soybean intercropping, and 28%, 24%, and 32% higher than those of maize/soybean intercropping, respectively, indicating stronger land-use and nutrient-uptake efficiency advantages under arid-irrigated conditions.

### 3.4. Potential for Resource Economy

Potential input economy, quantified as the theoretical N and P fertilizer requirements and estimated fertilizer-reduction potential of each intercropping system based on LER_N_ and LER_P_ values, was significantly affected by cropping systems, while years had non-significant effects on N and P requirements and potential reductions, and their interactions between cropping systems and years for estimated N and P requirements and potential N and P reductions were not significant (Supplementary Table S14). Based on observed LER_N_ values, cotton/soybean, maize/soybean, and sugarcane/soybean intercropping would theoretically require 190.8 kg N ha^−1^, 222.5 kg N ha^−1^, and 178.9 kg N ha^−1^, representing potential reductions of 36%, 26%, and 40%, respectively, compared to the N required by cotton, maize, and sugarcane in SCs (Table 4). Similarly, based on observed LER_P_ values, cotton/soybean, maize/soybean, and sugarcane/soybean intercropping would theoretically require 70.6 kg P ha^−1^, 68.2 kg P ha^−1^, and 73.0 kg P ha^−1^, representing potential reductions of 33%, 20%, and 39%, respectively, compared to the P required by cotton, maize, and sugarcane under SCs (Table 4). Among SBIs, sugarcane/soybean intercropping showed the highest theoretical N and P fertilizer-saving potential, with estimated N and P savings 11% and 36% higher than those in cotton/soybean intercropping and 56% and 180% higher than those in maize/soybean intercropping, respectively.

Moreover, cropping systems and years had significant effects on potential fertilizer cost savings for N and P, while their interactions were non-significant on potential fertilizer cost savings for N but significant on potential fertilizer cost savings for P; in addition, cropping systems, years, and their interactions had significant effects on potential total fertilizer cost savings (Supplementary Table S14). On average, compared to sole cotton, maize, and sugarcane cropping systems, cotton/soybean, maize/soybean, and sugarcane/soybean intercropping could potentially save 115.5 USD ha^−1^, 68.3 USD ha^−1^, and 144.2 USD ha^−1^, respectively (Table 4), if fertilizer inputs were reduced in proportion to the observed LER_N_ and LER_P_ values without yield loss.

### 3.5. Economic Profitability

Average total production cost across the four years was 1261 USD ha^−1^ for cotton/soybean intercropping, 1346 USD ha^−1^ for maize/soybean intercropping, and 1469 USD ha^−1^ for sugarcane/soybean intercropping, which was 22%, 19%, and 14% higher than the total production cost for main crops in sole cropping systems: cotton (1034 USD ha^−1^), maize (1132 USD ha^−1^), and sugarcane (1288 USD ha^−1^), respectively (Supplementary Tables S4 and S5). Despite the higher total production costs, all ICs consistently achieved higher net income and BCR values than their respective SCs, primarily due to the additional income from intercropped soybean, which only utilized the available and applied resources of the main crops under ICs. For instance, on average, the total net income and BCR of cotton/soybean, maize/soybean, and sugarcane/soybean intercropping were 1098 USD ha^−1^ and 1.9, 1084 USD ha^−1^ and 1.8, and 2937 USD ha^−1^ and 3.0, respectively, which were 154% and 33%, 29% and 4%, and 36% and 12% higher than the net income and BCR values of sole cotton, sole maize, and sole sugarcane, respectively (Table 5). Moreover, soybean intercropping with cotton, maize, and sugarcane consistently increased labor demand, expressed as person-days ha^−1^, as shown in Supplementary Tables S4 and S5. For example, averaged across the four years, labor requirement increased from 141 to 180 person-days ha^−1^ in cotton/soybean intercropping, from 135 to 164 person-days ha^−1^ in maize/soybean intercropping, and from 146 to 172 person-days ha^−1^ in sugarcane/soybean intercropping compared with their corresponding sole main-crop systems. Collectively, these results indicate that soybean intercropping with main crops increased net farm income and labor demand, expressed as person-days ha^−1^. In addition, the LER_N_- and LER_P_-based calculations suggest potential fertilizer cost savings if fertilizer inputs are adjusted in future reduced-fertilizer management scenarios; however, these fertilizer cost reductions were not directly tested in the present experiment.

## 4. Discussion

### 4.1. Yield Performance of Soybean-Based Intercropping Systems

Crop combination is a key factor in achieving higher crop yields and resource-use efficiency in ICs [16]. The spatial and temporal niche differentiation in intercrops, driven by variations in phenological development and growth dynamics, largely regulates resource capture through below- and above-ground interactions [23]. Unlike SCs, where crops share the same resource niches leading to increased competition for light, water, and nutrients, intercropping mitigates these competitive effects by capitalizing on interspecific complementarities and niche differentiation [4]. However, ICs including crops with asynchronous growth durations, e.g., cotton/soybean and sugarcane/soybean, demonstrate superior resource partitioning, thereby reducing interspecific competition and enhancing overall system’s productivity [18,24]. In line with previous reports, our results suggest that crop combinations and growth-stage differences in SBIs may have improved resource partitioning and reduced direct competition between component crops, thereby contributing to higher land productivity [10,11,14]. However, because root traits, canopy light interception, and below-ground interactions were not directly measured, these mechanisms should be interpreted as plausible explanations rather than directly demonstrated processes. The relative yields of main crops were lower in intercropping than in SCs [19,27,35]; however, this yield penalty was fully offset by the compensatory yield of intercropped soybean, which efficiently utilized residual resources from the main crops without requiring additional inputs, e.g., water or fertilizers [24]. These productivity advantages were evident as (a) each intercrop was planted at 100% of its sole planting density, resulting in a total planting density of 200% in all ICs [19], and (b) each intercrop received only 50% of the available land and applied resources compared to what it would have in SCs, yet each IC achieved a higher total LER value for land, N, and P [36].

Yield variability among intercrops highlights the importance of interspecies interactions, which ultimately regulate resource capture and sharing mechanisms under ICs [7,37]. Intercropped cotton and sugarcane exhibited yield reductions of 20% and 12%, respectively, while intercropped maize showed a 26% yield reduction. Similarly, intercropped soybean exhibited a lower yield reduction when grown with sugarcane 17% compared to when intercropped with cotton 28% and maize 41%. An important consideration in understanding our results is that soybean row spacing differed between sole cropping (50 cm) and intercropping systems (30 cm in cotton/soybean, 40 cm in sugarcane/soybean, and 50 cm in maize/soybean). However, soybean plant density was held constant at 167,000 plants ha^−1^ across all systems by adjusting within-row spacing accordingly. This approach ensures that differences in soybean performance are attributable to intercropping effects and resource competition/complementarity, not to population differences. The different row spacings in intercropping systems reflect locally optimized configurations that accommodate the main crop’s row spacing while maintaining constant soybean density [14,24,25]. This design choice prioritizes practical relevance and adoptability by farmers, as these configurations represent actual farmer practices in the region. The superior yield performance of intercrops in sugarcane/soybean and cotton/soybean intercropping compared to intercrops in maize/soybean intercropping can be attributed to morphological and physiological traits controlling resource utilization efficiencies [7,20,24]. Sugarcane and cotton, with their extended hierarchical canopy structures and vegetative phases, facilitate stratified light capture and complement below-ground nutrient acquisition [14,19], fostering an accommodating growth environment [38,39]. Another possible reason for these results is the slow growth patterns of cotton and sugarcane, which create a temporal advantage for intercropped soybean for resource exploitation and utilization in ICs [40,41,42]. During the initial growth phase, the slower canopy development of sugarcane and cotton may have allowed soybean to access more open inter-row space and available resources, which could partly explain its higher dry matter accumulation and yield performance in these systems [43,44]. In the later stages, soybean residues or rhizosphere effects may have contributed to nutrient cycling, but these processes were not directly measured in the present study. Therefore, their role should be regarded as a possible explanation that requires confirmation through measurements of root traits, biological nitrogen fixation, soil nutrient dynamics, and microbial activity [42,45]. In contrast, in maize/soybean ICs, the greater overlap between vegetative and reproductive growth stages may have intensified competition for space, light, and nutrients, which could explain the comparatively lower soybean yield, LER, and economic advantage observed in this system [46,47,48]. Collectively, these results indicate that the additive soybean-based intercropping systems improved land-use and economic performance compared with the corresponding sole main-crop systems. However, these advantages should not be attributed solely to interspecific complementarity, because the design maintained the full main-crop density while adding soybean as an additional crop. Thus, the observed improvements likely resulted from both increased total crop density/additional soybean production and possible temporal or spatial complementarity between the component crops. Nevertheless, differential yield responses across ICs reaffirm the necessity of strategic crop pairing based on growth stage synchronization and interspecific complementarities to maximize productivity and economic gains, particularly in resource-limited arid-irrigated regions. Moreover, a key conceptual distinction must be made when interpreting intercropping productivity metrics, particularly total system yield and LER. Total system yield simply sums the harvested product mass of the component crops within each intercropping system; however, this approach has limitations because it combines crop products with different physical properties, moisture contents, economic values, and end uses, such as seed cotton, maize grain, soybean seed, and sugarcane cane yield. In contrast, total LER measures land-use efficiency by comparing the land area required under sole cropping to produce the same combined outputs as intercropping. Therefore, total system yield can be lower than the yield of a sole crop while total LER remains greater than 1, because LER accounts for each crop’s productivity relative to its sole counterpart. For instance, maize/soybean intercropping produced 13% lower total system yield than sole maize (9.0 vs. 10.4 t ha^−1^), yet its LER of 1.33 indicates that producing the same maize and soybean outputs separately would require 33% more land. Economic indicators provide a third perspective by converting diverse crop outputs into a common monetary basis. Despite lower total system yield, maize/soybean intercropping produced 29% greater net income than sole maize. Therefore, total system yield, LER, monetary advantage index, net income, and BCR should be interpreted together, with greater emphasis on LER and value-based indicators when comparing diversified intercropping systems.

### 4.2. Resource Utilization Efficiency and Potential Input Economy Under Soybean-Based Intercropping Systems

Cotton, maize, and sugarcane are the main cash crops in arid-irrigated regions, where farmers typically apply excessive fertilizer inputs to maximize yield potential [7,38,44]. Cotton and sugarcane have longer growth durations than maize and require greater fertilizer inputs during later developmental stages, which begin around 80 days after cotton germination or 100 days after sugarcane sprouting, compared to their early growth stages [40,49]. However, traditional agronomic practices involve applying a disproportionate share of fertilizers—around 30–40% of N, 70–80% of P, and 100% of K—at the time of sowing [50,51]. The slow initial growth of cotton and sugarcane, compared to cereals and legumes, raises economic return and environmental concerns, particularly in SCs, where a substantial proportion of applied nutrients may remain unutilized during early growth stages, resulting in low nutrient utilization efficiency [14,49,52,53]. This misalignment between nutrient supply and crop demand aggravates environmental concerns, e.g., N leaching and P runoff, alongside economic inefficiencies due to increased input costs [24,38]. Thus, optimizing nutrient uptake and recycling during the critical 80–100-day period of slow growth in cotton and sugarcane is essential for mitigating nutrient losses and improving overall fertilizer use efficiency [54,55]. In this context, soybean intercropping, with a growth duration of 100–120 days within the interrow spaces of cotton and sugarcane, represents a strategic agroecological intervention [41,43]. Thus, soybean inclusion in these systems may have improved system-level nutrient uptake through more complementary crop growth patterns and resource use. However, rhizospheric interactions and interspecific facilitation were not directly measured and should therefore be interpreted as possible mechanisms [23,31]. This approach can produce additional legume crops while maintaining or slightly reducing the main crop yields, without the need for extra land dedicated to legume production, as observed in this study over four years and verified by previous research on SBIs [6,18,24,42,43]. Specifically, in this study, the farmer applied the same amount of N and P fertilizers as used in SCs, yet all ICs achieved higher total LER values for N and P. This indicates that the intercrops achieved higher system-level nutrient uptake efficiency under the same fertilizer input regime. Therefore, the observed LER_N_ and LER_P_ values suggest potential fertilizer-saving opportunities, but they do not demonstrate actual fertilizer reductions because all intercropping systems received the same fertilizer rates as their respective sole systems. The higher LER_N_ and LER_P_ values may be associated with interspecific facilitation, temporal niche differentiation, and complementary nutrient use between soybean and the main crops. Nevertheless, because biological nitrogen fixation, residual soil N and P, root interactions, and nutrient losses were not directly measured, the present data cannot identify the exact mechanisms responsible for these nutrient-use advantages [10,38,41]. While our estimates indicate substantial potential for fertilizer reduction, the actual implementation of reduced rates would require careful consideration of yield risk. A qualitative sensitivity analysis (Supplementary Table S6) examines the potential yield risk under different fertilizer reduction scenarios (10%, 20%, and 30%) based on the observed LER_N_ and LER_P_ values and temporal niche differentiation of each system. This analysis suggests that the sugarcane/soybean intercropping, with its higher LER values for N and P (1.61–1.70) and greater temporal niche differentiation, would likely tolerate greater fertilizer reduction (10–15%) with lower yield risk. In contrast, the maize/soybean intercropping (LER_N_ 1.35, LER_P_ 1.25) shows moderate to very high risk even at 10–20% reduction due to synchronous growth creating stronger competition for nutrients. The cotton/soybean system shows an intermediate risk profile. This qualitative assessment suggests that a cautious approach to fertilizer reduction might involve initial decreases of 7–10% below sole crop rates across all systems, with system-specific adjustments based on monitoring. Future research should test stepped fertilizer reduction treatments (e.g., 10%, 20%, 30% reductions) to identify optimal rates that balance input economy with yield stability in each intercropping system and validate the theoretical savings estimated in this research. Because no actual reduced-fertilizer treatments were included, these LER_N_- and LER_P_-based estimates could not be validated using model-performance metrics such as MAE, RMSE, or NSE; future fertilizer-gradient experiments should be used to calibrate and validate these estimates.

Notably, among the tested ICs, cotton/soybean and sugarcane/soybean intercropping achieved the highest LER values, surpassing those observed in maize/soybean intercropping. The lower LER in maize/soybean intercropping likely stems from the comparable phenological durations of both crops (maize: 120 ± 5 days; soybean: 110 ± 5 days), leading to a high degree of temporal overlap in resource acquisition and, consequently, intensified interspecific competition [4,47]. In contrast, cotton/soybean and sugarcane/soybean systems benefited from greater niche differentiation and asynchronous resource utilization [24,42], mitigating competitive pressure and fostering a more efficient partitioning of available resources [14,17,55]. Additionally, maize hybrids, characterized by rapid vegetative growth and high nutrient demand during the first 70–80 days, impose significant competitive stress on soybean, particularly in terms of N and P uptake. Conversely, soybean intercropped with cotton and sugarcane may have experienced relatively lower competitive pressure because of the slower early growth of these main crops, which could have favored soybean growth and nutrient uptake. However, direct measurements of root proliferation and nutrient acquisition pathways are needed to confirm this explanation [38,55]. Overall, these findings suggest the role of temporal complementarity and niche partitioning in optimizing resource-use efficiency within SBIs [12,17]. Under furrow irrigation, intercropped soybean received no separate irrigation beyond that supplied to the associated main crop, thereby avoiding additional water input to the soybean component. Differences in crop growth duration and canopy development may have influenced water competition and complementarity [56,57]; for example, cotton/soybean and sugarcane/soybean may have had less overlap in peak water demand than maize/soybean. However, soil moisture dynamics, root-zone water distribution, evapotranspiration, and water-use efficiency were not directly measured. Therefore, these water-related explanations should be interpreted as possible mechanisms and require confirmation in future studies. In addition to nutrient uptake, soybean-based intercropping may also influence soil physical structure over time, particularly under sandy loam soils in arid-irrigated systems. Diverse crop root systems can differ in rooting depth and density, which may affect soil moisture content in different soil layers [58], pore continuity, infiltration, and resistance to compaction. In the present study, cotton, maize, sugarcane, and soybean differed strongly in growth duration and root architecture; therefore, their intercropping may have altered below-ground spatial occupation and soil structural dynamics compared with sole cropping. However, soil physical properties after intercropping, such as aggregate stability, porosity, infiltration rate, penetration resistance, and post-harvest bulk density, were not directly measured. Thus, the present results cannot confirm whether the observed agronomic advantages were associated with changes in soil physical structure. Future studies should include direct measurements of soil structure and root distribution to clarify how long-term soybean-based intercropping affects soil physical quality under arid-irrigated conditions. Altogether, these insights provide a useful foundation for designing region-specific ICs that can improve farm productivity and indicate potential input-economy benefits, although fertilizer-reduction and environmental effects require direct validation.

### 4.3. Economic Returns of Soybean-Based Intercropping Systems

Adoption of any new innovation in agricultural production hinges on its economic viability and profitability, particularly in resource-intensive farming systems, where farmers are keenly focused on achieving high economic returns [18,24]. Given the risk-averse nature of farming communities, particularly in regions with volatile agricultural markets and price fluctuations, sole cropping remains dominant due to its yield and market stability [2,16,59]. However, ICs that enhance gross margin, land-use efficiency, and labor demand can serve as a sustainable intensification strategy, particularly for smallholder farmers who rely on agriculture for both household food security and income generation [6,59]. Our findings showed that soybean intercropping with cotton, maize, and sugarcane improved net income, labor requirement, and overall system productivity compared with their respective SCs. Specifically, soybean intercropping with cotton, maize, and sugarcane increased total net income by 154%, 29%, and 36% and labor requirement by 28%, 21%, and 18%, respectively, relative to SCs, indicating that soybean intercropping with main crops increased farm incomes but also required greater labor input, expressed as person-days ha^−1^, than the corresponding sole cropping systems [2,59]. The observed increase in net profit was primarily attributed to the additional soybean yield, which was produced without extra capital investment in irrigation, land preparation, or fertilizer inputs [24]. Furthermore, the value-based indices presented in Supplementary Table S15 also confirm the economic advantage of the intercropping system over sole cropping. The monetary advantage index ranged from +603 USD ha^−1^ in maize/soybean to +1829 USD ha^−1^ in sugarcane/soybean, indicating that intercropping consistently generated greater gross value than sole cropping despite occasionally lower total system mass. The inclusion of an extra crop within the cropping sequence increased agricultural labor demand, which may be beneficial for employment generation in labor-surplus regions but may also represent an additional input cost and management burden for farmers. In regions facing labor shortages, rising wage rates, or competition for labor from off-farm employment, this increased labor requirement could become a barrier to adoption. Therefore, while soybean-based intercropping showed clear economic advantages under the tested farmer-field conditions, broader adoption will require further socio-economic assessment of labor availability, mechanization feasibility, market access, and farmer decision-making.

### 4.4. Limitations and Future Research Needs

While this four-year farmer-field study provides useful field-based evidence for the agronomic and economic performance of SBIs under the tested conditions, several limitations should be acknowledged when interpreting the results. First, the tested systems followed an additive intercropping design, in which the main crop density was maintained and soybean was added as an additional crop. Therefore, the observed improvements in land-use efficiency, nutrient uptake, and economic returns may reflect the combined effects of additional soybean production, increased total plant density, and possible crop complementarity, rather than complementarity alone. Second, the fertilizer-reduction estimates are theoretical, as the experiment did not include treatments with reduced fertilizer rates; therefore, validation studies with step-wise fertilizer reduction are needed. Third, the research was conducted at a single location with specific soil and climate conditions, crop varieties, annual sugarcane planting, and planting configurations, which may limit generalizability. Fourth, the study did not directly measure biological nitrogen fixation, residual soil N and P, nutrient losses through leaching or runoff, root traits, rhizosphere processes, microbial activity, soil physical structure, soil moisture dynamics, root-zone water distribution, evapotranspiration, water-use efficiency, or long-term soil health changes. Thus, mechanistic explanations related to nutrient facilitation, rhizospheric interactions, nutrient mineralization, and environmental benefits should be interpreted as possible explanations rather than directly demonstrated processes. Fifth, the economic analysis was based on local market prices, labor wages, input costs, and exchange rates, which varied across years. Thus, absolute profit values may differ under different market conditions. In addition, the higher labor requirement in intercropping systems may be beneficial for employment generation in labor-surplus regions but could limit adoption in areas with labor shortages or high wage rates. Hence, future research should: (i) validate these results across multiple locations and contrasting soil and climatic conditions; (ii) test different crop varieties, row arrangements, and planting densities; (iii) evaluate intercropping performance under ratoon sugarcane management; (iv) include factorial fertilizer treatments with actual N and P reductions; (v) directly measure biological nitrogen fixation, nutrient losses, root and rhizosphere processes, microbial activity, soil physical structure, soil moisture dynamics, root-zone water distribution, evapotranspiration, water-use efficiency, and long-term soil health; and (vi) develop and evaluate mechanized solutions to reduce labor dependence and improve scalability.

## 5. Conclusions

Available evidence from this four-year farmer-field study shows that soybean-based intercropping with cotton, maize, and sugarcane can improve land productivity, nutrient-uptake efficiency, and farm profitability under arid-irrigated conditions. Across the tested intercropping systems, total LER_L_, LER_N_, and LER_P_ values ranged from 1.33 to 1.71, 1.35–1.68, and 1.25–1.64, respectively, indicating clear land-use and nutrient-uptake advantages compared with sole cropping. These benefits were particularly evident in cotton/soybean and sugarcane/soybean intercropping, where greater temporal niche differentiation between soybean and the main crop supported higher resource-use efficiency and economic returns. The LER_N_- and LER_P_-based calculations also suggest potential fertilizer input-economy benefits; however, these estimates are theoretical and require validation through field experiments with actual reduced-fertilizer treatments. The results further indicate an important labor-related trade-off: soybean-based intercropping increased labor demand, which may support employment generation in labor-surplus regions but could hinder adoption where labor shortages or high wage rates exist. Therefore, while soybean-based intercropping offers a promising option for sustainable intensification under conditions similar to those tested here, broader adoption will depend on farmer preferences, labor availability, mechanization options, market access, and extension support. Future studies should combine multi-location agronomic trials, reduced-fertilizer treatments, ratoon sugarcane evaluation, long-term soil and water measurements, mechanization assessment, and socio-economic adoption surveys to better evaluate the scalability of these systems.

## Figures and Tables

**Table 1:** Dry matter (t ha^−1^), nitrogen uptake (kg ha^−1^), phosphorus uptake (kg ha^−1^), and crop yield (t ha^−1^) of main crops (cotton, maize, and sugarcane) under sole and intercropping systems.

Years	Crop Indices	Cotton		Maize		Sugarcane	
		ICs	SCs	ICs	SCs	ICs	SCs
2021	Dry matter	8.5 b	10.6 a	15.7 b	20.1 a	25.2 b	28.8 a
	Nitrogen uptake	187.5 b	234.6 a	186.3 b	257.6 a	211.0 b	2428 a
	Phosphorus uptake	30.3 b	41.98 a	26.3 b	37.9 a	37.9 b	44.3 a
	Crop yield	1.2 b	1.5 a	7.4 b	10.1 a	77.9 b	87.8 a
2022	Dry matter	8.7 b	10.4 a	16.1 b	19.8 a	26.1 b	29.4 a
	Nitrogen uptake	182.2 b	221.5 a	192.9 b	263.8 a	218.7 b	254.8 a
	Phosphorus uptake	33.1 b	39.7 a	27.3 b	38.2 a	35.7 b	42.1 a
	Crop yield	1.3 b	1.6 a	7.6 a	10.3 b	79.1 b	90.5 a
2023	Dry matter	9.5 b	10.9 a	16.1 b	21.1 a	27.7 b	30.9 a
	Nitrogen uptake	199.6 b	242.4 a	183.2 b	254.1 a	226.6 b	269.4 a
	Phosphorus uptake	34.4 b	43.6 a	28.2 b	40.2 a	40.6 b	45.6 a
	Crop yield	1.4 b	1.7 a	7.8 b	10.6 a	78.9 b	91.7 a
2024	Dry matter	9.8 b	11.3 a	16.9 b	22.5 a	28.3 b	31.6 a
	Nitrogen uptake	212.3 b	257.1 a	198.1 b	269.7 a	244.2 b	275.7 a
	Phosphorus uptake	35.6 b	45.3 a	27.6 b	39.6 a	42.3 b	47.8 a
	Crop yield	1.3 b	1.6 a	7.8 a	10.4 b	80.5 b	89.4 a
Mean	Dry matter	9.1	10.8	16.3	20.9	26.8	30.2
	Nitrogen uptake	195.4	238.9	190.1	261.3	225.1	260.4
	Phosphorus uptake	33.4	42.6	27.4	39.0	39.2	44.9
	Crop yield	1.3	1.6	7.7	10.4	79.1	89.9

ICs represent main crops in cotton/soybean, maize/soybean, and sugarcane/soybean intercropping systems, while SCs denote crops in sole cropping systems. Note: Values are means across three replications. Different lowercase letters within the same year and crop indicate significant differences among cropping systems at *p* < 0.05. Four-year means are provided to summarize the overall treatment response.

**Table 2:** Dry matter (t ha^−1^), nitrogen uptake (kg ha^−1^), phosphorus uptake (kg ha^−1^), and crop yield (t ha^−1^) of soybean under sole and intercropping systems.

Years	Crop Indices	SI_C_	SI_M_	SI_Su_	SCs
2021	Dry matter	2.4 c	2.1 d	2.8 b	3.1 a
	Nitrogen uptake	135.4 b	107.7 c	139.3 b	168.5 a
	Phosphorus uptake	18.2 c	14.1 d	19.3 b	25.6 a
	Crop yield	1.4 bc	1.2 c	1.7 b	2.2 a
2022	Dry matter	2.5 c	2.2 d	3.1 b	3.4 a
	Nitrogen uptake	146.2 b	126.5 c	160.9 b	208.2 a
	Phosphorus uptake	20.1 c	14.7 d	22.1 b	28.6 a
	Crop yield	1.7 bc	1.4 c	1.9 b	2.2 a
2023	Dry matter	2.8 b	2.4 c	3.3 a	3.6 a
	Nitrogen uptake	142.1 b	113.8 c	153.5 b	184.3 a
	Phosphorus uptake	19.3 c	15.8 d	21.6 b	27.5 a
	Crop yield	1.5 b	1.2 c	1.7 b	2.2 a
2024	Dry matter	3.1 c	2.7 d	3.5 b	3.8 a
	Nitrogen uptake	159.5 b	133.3 c	176.4 b	215.8 a
	Phosphorus uptake	21.1 c	16.1 d	23.2 b	29.9 a
	Crop yield	1.7 b	1.4 c	2.1 a	2.4 a
Mean	Dry matter	2.7	2.4	3.2	3.5
	Nitrogen uptake	145.8	120.4	157.5	194.2
	Phosphorus uptake	19.7	15.2	21.6	27.9
	Crop yield	1.6	1.3	1.8	2.2

SI_C_, SI_M_, and SI_Su_ represent soybean intercropped with cotton, maize, and sugarcane, respectively in cotton/soybean, maize/soybean, and sugarcane/soybean intercropping systems, while SCs denote soybean in a sole cropping system. Note: Values are means across three replications. Different lowercase letters within the same year and crop indicate significant differences among cropping systems at *p* < 0.05. Four-year means are provided to summarize the overall treatment response.

**Table 3:** Total system yield (TSY; t ha^−1^), total system nitrogen uptake (TSNU; kg ha^−1^), and total system phosphorus uptake (TSPU; kg ha^−1^) of cotton/soybean, maize/soybean, and sugarcane/soybean intercropping systems.

Years	Treatments	TSY	TSNU	TSPU
2021	Cotton/soybean	2.6 c	322.9 b	48.5 b
	Maize/soybean	8.6 b	294.0 c	40.5 c
	Sugarcane/soybean	79.6 a	350.2 a	57.3 a
2022	Cotton/soybean	3.0 c	328.4 b	53.2 b
	Maize/soybean	9.1 b	319.4 b	42.0 c
	Sugarcane/soybean	81.0 a	379.6 a	57.8 a
2023	Cotton/soybean	2.9 c	341.7 b	53.7 b
	Maize/soybean	9.1 b	297.0 c	43.9 c
	Sugarcane/soybean	80.5 a	380.1 a	62.2 a
2024	Cotton/soybean	3.1 c	371.8 b	56.7 b
	Maize/soybean	9.2 b	331.3 c	43.7 c
	Sugarcane/soybean	82.7 a	420.7 a	65.6 a
Mean	Cotton/soybean	2.9	341.2	53.0
	Maize/soybean	9.0	310.4	42.5
	Sugarcane/soybean	81.0	382.6	60.7

Means within a column that do not share the same letter differ significantly at *p* ≤ 0.05, based on the least significant difference test, calculated separately for each crop and year, and values are means across three replications. Note: Total system yield represents the mass-based sum of the main crop yield and soybean yield within each intercropping system. Because this value combines different crop products, it should be interpreted as a descriptive indicator and considered together with LER and economic indicators.

**Table 4:** Estimated nitrogen requirement (ENR; kg ha^−1^), estimated phosphorus requirement (EPR; kg ha^−1^), potential nitrogen reduction (PNR; kg ha^−1^), potential phosphorus reduction (PPR; kg ha^−1^) based on LER_N_ and LER_P_ values, potential cost savings on nitrogen (PCS_N_; USD ha^−1^), potential cost savings on phosphorus (PCS_P_; USD ha^−1^), and potential total cost savings on nitrogen and phosphorus fertilizers (PTCS_NP_; USD ha^−1^) of soybean-based intercropping systems.

Years	Treatments	ENR	EPR	PNR	PPR	PCS_N_	PCS_P_	PTCS_NP_ *
		Main Crop	Soybean	Main Crop	Soybean	Main Crop	Soybean	
2021	Cotton/soybean	187.0 b	73.3 a	113.0 a	31.7 b	60.3 a	67.7 b	127.9 b
	Maize/soybean	220.1 a	68.2 b	79.9 b	16.8 c	42.6 b	35.8 c	78.5 c
	Sugarcane/soybean	176.5 b	74.5 a	123.5 a	45.5 a	65.9 a	97.2 a	163.1 a
2022	Cotton/soybean	196.4 b	68.2 b	103.6 a	36.8 b	44.2 a	62.7 b	106.9 b
	Maize/soybean	224.2 a	69.1 b	75.8 b	15.9 c	32.2 b	27.1 c	59.4 c
	Sugarcane/soybean	183.9 b	73.9 a	116.1 a	46.1 a	49.5 a	78.6 a	128.1 a
2023	Cotton/soybean	188.0 b	70.4 a	112.0 b	34.6 b	42.0 b	51.8 b	93.8 b
	Maize/soybean	223.9 a	66.7 b	76.1 c	18.3 c	28.5 c	27.4 c	55.9 c
	Sugarcane/soybean	179.3 c	71.6 a	120.7 a	48.4 a	45.3 a	72.6 a	117.8 a
2024	Cotton/soybean	191.5 b	70.3 ab	108.5 b	34.7 b	68.1 b	65.3 b	133.4 b
	Maize/soybean	222.0 a	68.8 b	78.0 c	16.2 c	48.9 c	30.4 c	79.4 c
	Sugarcane/soybean	176.0 a	72.2 a	124.0 a	47.8 a	77.8 a	90.1 a	167.9 a
Mean	Cotton/soybean	190.8	70.6	109.2	34.4	53.6	61.9	115.5
	Maize/soybean	222.5	68.2	77.5	16.8	38.1	30.2	68.3
	Sugarcane/soybean	178.9	73.0	121.1	47.0	59.6	84.6	144.2

The cotton/soybean, maize/soybean, and sugarcane/soybean represent three different soybean-based intercropping systems. Means within a column that do not share the same letter differ significantly at *p* ≤ 0.05, based on the least significant difference test, calculated separately for each crop and year, and values are means across three replications. All values are theoretical estimates based on LER_N_ and LER_P_ calculations. * The total cost savings on nitrogen and phosphorus is the sum of the main crop’s cost savings on nitrogen and phosphorus and the soybean’s cost savings on nitrogen and phosphorus in cotton/soybean, maize/soybean, and sugarcane/soybean intercropping systems. Note: Estimated fertilizer requirements, potential fertilizer reductions, and potential cost savings were calculated theoretically from observed LER_N_ and LER_P_ values. These values represent potential input-economy indicators and should not be interpreted as experimentally validated fertilizer reductions.

**Table 5:** Gross income, net income, and benefit-to-cost ratio (BCR) of different intercropping and sole cropping systems in 2021, 2022, 2023, and 2024. All values are given in USD ha^−1^.

Treatments	Gross Income				Mean	Net income				Mean	BCR				Mean
	2021	2022	2023	2024		2021	2022	2023	2024		2021	2022	2023	2024	
Cotton/soybean	2332	2210	2192	2703	2359	1064	964	1143	1454	1156	1.8	1.8	2.1	2.2	2.0
Maize/soybean	2135	2454	2183	2949	2430	717	1068	1001	1551	1084	1.5	1.8	1.8	2.1	1.8
Sugarcane/soybean	4695	4083	4009	4837	4406	3101	2584	2721	3342	2937	2.9	2.7	3.1	3.2	3.0
Sole cotton	1776	1482	1230	1377	1466	697	425	290	317	432	1.6	1.4	1.3	1.3	1.4
Sole maize	1868	2120	1644	2267	1975	669	968	641	1094	843	1.6	1.8	1.6	1.9	1.7
Sole sugarcane	4042	3350	3164	3226	3445	2630	2046	2029	1922	2157	2.9	2.6	2.8	2.5	2.7
Sole soybean	1398	1359	1709	2130	1649	516	488	995	1306	826	1.6	1.6	2.4	2.6	2.0

The cotton/soybean, maize/soybean, and sugarcane/soybean represent three different soybean-based intercropping systems. Each year, the market prices for maize and soybeans were obtained in June, for cotton in September, and for sugarcane in January. The market price for cotton was USD 1200 t^−1^ in 2021, 919 t^−1^ in 2022, 707 t^−1^ in 2023, and 848 t^−1^ in 2024; for maize, it was 184 t^−1^ in 2021, 206 t^−1^ in 2022, 155 t^−1^ in 2023, and 217 t^−1^ in 2024; for sugarcane, it was 46 t^−1^ in 2022, 37 t^−1^ in 2023, 34 t^−1^ in 2024, and 36 t^−1^ in 2025; and for soybean, it was USD 650 t^−1^ in 2021, 615 t^−1^ in 2022, 776 t^−1^ in 2023, and 903 t^−1^ in 2024. Note: Gross income, net income, and BCR were calculated using year-specific crop prices and exchange rates. Net income was calculated as gross income minus total expenditure, and BCR was calculated as gross income divided by total expenditure.

## Data Availability

The original contributions presented in the study are included in the article/Supplementary Material, further inquiries can be directed to the corresponding author.

## Supplementary Materials

The following supporting information can be downloaded at: https://www.mdpi.com/article/10.3390/plants15142111/s1.
