# Supplementary material for: Yield Performance, Resource-Use Efficiency, and Economic Profitability from Adopting Soybean-Based Cotton/Maize/Sugarcane Intercropping Systems Under Arid-Irrigated Conditions
Source: Plants (Basel). 2026 Jul 8;15(14):2111. doi: 10.3390/plants15142111 (PMC13416271; doi:10.3390/plants15142111)
Supplement: Supplementary file 1 [file plants-15-02111-s001.zip › plants-4395300-supplementary.pdf]

Supplementary Table S1
Complete planting configurations for cultivating main crops (cotton/maize/sugarcane) and soybean under sole and intercropping systems.

Treatments *	Row distance (cm)		Row ratio	Strip distance (cm) **	Plant/bud distance (cm)		Plant/bud density (m^{-2})		Total density (m^{-2})
	Main crop	Soybean	Main crop: Soybean		Main crop	Soybean	Main crop	Soybean***	
Cotton/soybean	90	30	1:2	30	22	20	05	17	22
Maize/soybean	50	50	2:2	50	11	06	09	17	26
Sugarcane/soybean	80	40	1:2	20	11	15	11	17	28
Sole cotton	90	--	--	--	22	--	05	--	05
Sole maize	50	--	--	--	22	--	09	--	09
Sole sugarcane	80	--	--	--	11	--	11	--	11
Sole soybean	--	50	--	--	--	12	--	17	17

* Cotton/soybean, maize/soybean, and sugarcane/soybean represent three different soybean-based intercropping systems. ** Strip distance refers to the distance between the strips of the main crops (cotton, maize, or sugarcane) and soybean under intercropping systems. *** Soybean plant density was maintained at 167,000 plants ha^{-1} across all systems by adjusting within-row spacing to compensate for different row widths. This ensures comparability of soybean performance between sole and intercropping systems.

Supplementary Table S2
Worked example showing calculation of potential nitrogen (N) and phosphorus (P) reduction for sugarcane/soybean intercropping (2021 data).

Step	Parameter	Value	Calculation
Step 1: Calculate pLER values from uptake	N uptake of sugarcane in intercropping	211 kg ha^{-1}	From Table 4
	N uptake of sugarcane in sole	242 kg ha^{-1}	From Table 4
	$pLER_N$ (sugarcane)	0.87	211 ÷ 242
	N uptake of soybean in intercropping	139 kg ha^{-1}	From Table 5
	N uptake of soybean in sole	169 kg ha^{-1}	From Table 5
	$pLER_N$ (soybean)	0.82	139 ÷ 169
Step 2: Calculate Total LER_N	Total LER_N	1.69	0.87 + 0.82
Step 3: Estimate theoretical N requirement	N fertilizer applied to sole sugarcane	300 kg ha^{-1}	From Section 2.2
	Theoretical N requirement for intercrop	177.5 kg ha^{-1}	300 ÷ 1.69
Step 4: Calculate potential N reduction	Potential N reduction (PNR)	122.5 kg ha^{-1}	300 − 177.5
Step 5: Repeat for phosphorus	P uptake of sugarcane in intercropping	37.9 kg ha^{-1}	From Table 4
	P uptake of sugarcane in sole	44.3 kg ha^{-1}	From Table 4
	$pLER_P$ (sugarcane)	0.86	37.9 ÷ 44.3
	P uptake of soybean in intercropping	19.3 kg ha^{-1}	From Table 5
	P uptake of soybean in sole	25.6 kg ha^{-1}	From Table 5
	$pLER_P$ (soybean)	0.75	19.3 ÷ 25.6
	Total LER_P	1.61	0.86 + 0.75
	P fertilizer applied to sole sugarcane	120 kg ha^{-1}	From Section 2.2
	Theoretical P requirement for intercrop	74.5 kg ha^{-1}	120 ÷ 1.61
	Potential P reduction (PPR)	45.5 kg ha^{-1}	120 − 74.5

Note: Values may differ slightly from Table 8 averages due to rounding. This example uses 2021 data of sugarcane/soybean intercropping system; Table 8 presents 4-year averages. All values are theoretical estimates based on LER calculations for N and P; actual reduced fertilizer treatments were not tested in this experiment.

Supplementary Table S3
Assumptions underlying theoretical fertilizer reduction estimates.

Assumption	Explanation	Justification
Proportionality between uptake efficiency and fertilizer use efficiency	LER_N and LER_P values reflect relative nutrient uptake efficiency, which is assumed to be proportional to how efficiently fertilizer could be used	This is the standard interpretation of LER for N and P in intercropping research (Gou et al., 2018)
Similar soil nutrient contributions across systems	Native soil N and P, mineralization rates, and atmospheric deposition are assumed comparable between sole and intercropping systems	All treatments (sole and intercropping) were conducted in the same field with uniform soil conditions
Biological nitrogen fixation reflected in total N uptake	Biological nitrogen fixation by soybean is not measured separately but is included in total plant N accumulation	Total N uptake represents the actual N the crop acquired from all sources under sole and intercropping
Proportionality holds across reduction range	The relationship between uptake and fertilizer requirement is assumed linear across the reduction scenarios considered	This is a simplifying assumption for theoretical estimates; validation requires stepped reduction experiments

Note: These assumptions are necessary for theoretical estimation. The authors acknowledge that actual field conditions involve complex interactions, and therefore present these estimates as potentials requiring experimental validation.

Supplementary Table S4

Year-wise costs of inputs, field operations, and labor, together with working days and labor requirement, for cotton, maize, sugarcane, and soybean under sole cropping systems.

Input/field operation (a)	Cost USD (ha^{-1}) *																			
	Cotton				Average	Maize				Average	Sugarcane				Average	Soybean				Average
	2021	2022	2023	2024		2021	2022	2023	2024		2021	2022	2023	2024		2021	2022	2023	2024	
Crop seed	23	24	21	27	24	182	170	153	187	173	152	133	102	107	123	38	36	34	45	38
Land preparation	106	109	102	98	104	106	109	102	98	104	106	109	102	98	104	106	109	102	98	104
Urea	160	160	180	226	181	160	160	180	226	181	160	160	180	226	181	43	43	48	60	48
Single super phosphate	181	181	153	167	171	181	181	153	167	171	256	256	216	235	241	181	181	153	167	171
Chemicals **	136	153	138	150	144	121	119	95	123	115	174	167	142	177	165	53	61	57	60	58
Irrigation	133	120	94	118	116	121	109	85	107	106	194	174	136	171	169	85	76	60	75	74
Labor cost ***	339	311	251	275	294	327	305	234	265	283	370	305	256	289	305	315	300	217	260	273
Total	1079	1057	939	1060	1034	1199	1152	1003	1173	1132	1412	1304	1134	1304	1288	882	871	714	824	823

Labor requirement (b)	Number/person-days (ha^{-1})																			
	Cotton				Average	Maize				Average	Sugarcane				Average	Soybean				Average
	2021	2022	2023	2024		2021	2022	2023	2024		2021	2022	2023	2024		2021	2022	2023	2024	
Working days	89	86	84	89	87	89	94	91	89	91	114	104	114	109	110	64	64	64	62	64
Labor requirement	138	141	146	138	141	133	138	136	133	135	151	138	148	146	146	128	136	126	131	130

* All prices were obtained in the local currency (Pakistani rupee, PKR) and converted into US dollars (USD) using the average exchange rate of each year. One USD was equivalent to PKR 163 in 2021, PKR 204 in 2022, PKR 290 in 2023, and PKR 277 in 2024. ** Chemicals include the costs of fungicides, weedicides, and insecticides. *** Labor costs include all expenses paid to laborers for tasks such as hand sowing of crops, irrigation and fertilizer application, weedicide and pesticide spraying, hand weeding in soybean, harvesting, threshing, and hand-picking of cotton.

Supplementary Table S5
Year-wise costs of inputs, field operations, and labor, together with working days and labor requirement, for cotton/soybean, maize/soybean, and sugarcane/soybean intercropping systems.

Input/field operation (a)	Cost USD (ha^{-1}) *														
	Cotton/soybean				Average	Maize/soybean				Average	Sugarcane/soybean				Average
	2021	2022	2023	2024		2021	2022	2023	2024		2021	2022	2023	2024	
Crop seed	61	61	55	71	62	220	206	187	232	211	189	170	136	152	162
Land preparation	106	109	102	98	104	106	109	102	98	104	106	109	102	98	104
Urea	160	160	180	226	181	160	160	180	226	181	160	160	180	226	181
Single super phosphate	181	181	153	167	171	181	181	153	167	171	256	256	216	235	241
Chemicals **	189	213	195	210	202	174	179	153	183	172	227	228	200	237	223
Irrigation	133	120	94	118	116	121	109	85	107	106	194	174	136	171	169
Labor cost ***	430	398	284	360	368	394	376	279	326	344	400	338	275	316	332
Total	1322	1307	1106	1309	1261	1417	1385	1182	1398	1346	1594	1499	1288	1494	1469
Labor requirement (b)	Number/person-days (ha^{-1})														
	Cotton/soybean				Average	Maize/soybean				Average	Sugarcane/soybean				Average
	2021	2022	2023	2024		2021	2022	2023	2024		2021	2022	2023	2024	
Working days	100	96	95	104	99	90	96	93	91	93	112	104	112	109	109
Labor requirement	175	180	182	182	180	161	170	162	164	164	175	168	172	174	172

* All prices were obtained in the local currency (Pakistani rupee, PKR) and converted into US dollars (USD) using the average exchange rate of each year. One USD was equivalent to PKR 163 in 2021, PKR 204 in 2022, PKR 290 in 2023, and PKR 277 in 2024. ** Chemicals include the costs of fungicides, weedicides, and insecticides for both crops under soybean-based intercropping systems. *** Labor costs include all expenses paid to laborers for tasks such as hand sowing of crops irrigation and fertilizer application, weedicide and pesticide spraying, hand weeding in soybean, harvesting, threshing, and hand-picking of cotton.

Supplementary Table S6
Qualitative sensitivity analysis: Potential yield risk under different fertilizer reduction scenarios in soybean-based intercropping systems under arid irrigated conditions.

Intercropping system	LER_N	LER_P	Low reduction (10%)	Moderate reduction (20%)	High reduction (30%)
Cotton/soybean	1.57	1.49	Low risk	Moderate risk	High risk
Maize/soybean	1.35	1.25	Moderate risk	High risk	Very high risk
Sugarcane/soybean	1.68	1.64	Low risk	Low-moderate risk	Moderate risk

Note: Risk assessment is qualitative, based on LER values for N and P for each intercropping system and temporal niche differentiation. Lower LER values and synchronous growth (maize/soybean) indicate lower buffering capacity against nutrient reduction.

Supplementary Table S7
List of mixed effects models fitted to data.

Models	Explanatory variables	Equations
1	Dry matter (DM), nitrogen uptake (NU), and phosphorus uptake (PU)	$(DM, NU, PU)i = \beta o + \beta_1 * Year_i + \beta_2 * CS_i + \beta_3 * Year_i * CS_i + \alpha_i + \varepsilon_i$
2	Crop yield (CY)	$(CY)i = \beta o + \beta_1 * Year_i + \beta_2 * CS_i + \beta_3 * Year_i * CS_i + \alpha_i + \varepsilon_i$
3	Total system yield (TSY), total system nitrogen uptake (TSNU), and total system phosphorus uptake (TSPU)	$(TSY, TSNU, TSPU)i = \beta o + \beta_1 * Year_i + \beta_2 * CS_i + \beta_3 * Year_i * CS_i + \alpha_i + \varepsilon_i$
4	Partial land equivalent ratios (pLER) and total equivalent ratios (total LER) for land, nitrogen, and phosphorus	$(pLER, total\ LER)i = \beta o + \beta_1 * Year_i + \beta_2 * CS_i + \beta_3 * Year_i * CS_i + \alpha_i + \varepsilon_i$
5	Nitrogen requirement (NR), phosphorus requirement (PR), nitrogen saving (NS), phosphorus saving (PS), cost savings on nitrogen (CSN), cost savings on phosphorus (CSP), and total cost savings on nitrogen and phosphorus fertilizers (TCSNP).	$(NR, PR, NS, PS, CSN, CSP, TCSNP)i = \beta o + \beta_1 * Year_i + \beta_2 * CS_i + \beta_3 * Year_i * CS_i + \alpha_i + \varepsilon_i$

Note: α_i was the random block effect, ε_i was the residual error, and α_i and ε_i were assumed to be normally distributed with constant variances.

Supplementary Table S8
Mixed model ANOVAs for the effects of years (Y) and cropping systems (CS) on dry matter of main crops (cotton, maize, and sugarcane) and soybean.

Source	Cotton/soybean				Maize/soybean				Sugarcane/soybean			
	Cotton		Soybean		Maize		Soybean		Sugarcane		Soybean	
	F	P	F	P	F	P	F	P	F	P	F	P
Y	3.79	**0.031***	20.37	**0.001**	2.96	0.064	20.48	**0.001**	0.90	0.464	15.70	**0.001**
CS**	44.28	**0.001**	17.70	**0.001**	88.86	**0.001**	13.07	**0.002**	5.55	**0.032**	19.36	**0.001**
Y × CS	0.29	0.836	0.67	0.581	0.55	0.653	0.55	0.654	0.003	0.99	0.09	0.965

* All significant values ($p < 0.05$) are marked bold.
** Cropping systems include crops, e.g., cotton, maize, sugarcane, and soybean, under soybean-based intercropping systems (cotton/soybean, maize/soybean, and sugarcane/soybean) and their respective sole cropping systems.

Supplementary Table S9
Mixed model ANOVAs for the effects of years (Y) and cropping systems (CS) on nitrogen uptake of main crops (cotton, maize, and sugarcane) and soybean.

Source	Cotton/soybean				Maize/soybean				Sugarcane/soybean			
	Cotton		Soybean		Maize		Soybean		Sugarcane		Soybean	
	F	P	F	P	F	P	F	P	F	P	F	P
Y	6.96	**0.003***	5.52	**0.009**	2.08	0.143	5.43	**0.009**	5.67	**0.008**	19.02	**0.001**
CS**	66.64	**0.001**	52.71	**0.001**	229.36	**0.001**	96.60	**0.001**	33.31	**0.001**	75.60	**0.001**
Y × CS	0.09	0.960	0.97	0.432	0.001	0.99	1.99	0.155	0.21	0.890	0.98	0.426

* All significant values ($p < 0.05$) are marked bold.
** Cropping systems include crops, e.g., cotton, maize, sugarcane, and soybean, under soybean-based intercropping systems (cotton/soybean, maize/soybean, and sugarcane/soybean) and their respective sole cropping systems.

Supplementary Table S10
Mixed model ANOVAs for the effects of years (Y) and cropping systems (CS) on phosphorus uptake of main crops (cotton, maize, and sugarcane) and soybean.

Source	Cotton/soybean				Maize/soybean				Sugarcane/soybean			
	Cotton		Soybean		Maize		Soybean		Sugarcane		Soybean	
	F	P	F	P	F	P	F	P	F	P	F	P
Y	11.48	**0.001***	2.30	0.116	6.72	**0.004**	12.83	**0.001**	21.41	**0.001**	5.26	**0.010**
CS**	226.15	**0.001**	67.08	**0.001**	182.96	**0.001**	707.35	**0.001**	103.06	**0.001**	70.52	**0.001**
Y × CS	2.95	0.064	0.087	0.966	0.565	0.646	8.85	**0.001**	0.395	0.758	0.05	0.987

* All significant values ($p < 0.05$) are marked bold.
** Cropping systems include crops, e.g., cotton, maize, sugarcane, and soybean, under soybean-based intercropping systems (cotton/soybean, maize/soybean, and sugarcane/soybean) and their respective sole cropping systems.

Supplementary Table S11
Mixed model ANOVAs for the effects of years (Y) and cropping systems (CS) on on total system yield (TSY), total system nitrogen uptake (TSNU) and total system phosphorus uptake (TSPU).

Source	TSY		TSNU		TSPU	
	F	P	F	P	F	P
Y	1.89	0.159	13.71	**0.001**	30.93	**0.001**
CS**	15259.85	**0.001***	50.51	**0.001**	425.71	**0.001**
Y × CS	0.778	0.595	1.04	0.427	3.33	**0.016**

* All significant values ($p < 0.05$) are marked bold.
** Cropping systems include three different soybean-based intercropping systems (cotton/soybean, maize/soybean, and sugarcane/soybean).

Supplementary Table S12
Mixed model ANOVAs for the effects of years (Y) and cropping systems (CS) on crop yield of main crops (cotton, maize, and sugarcane) and soybean.

Source	Cotton/soybean				Maize/soybean				Sugarcane/soybean			
	Cotton		Soybean		Maize		Soybean		Sugarcane		Soybean	
	F	P	F	P	F	P	F	P	F	P	F	P
Y	4.83	**0.014**	1.52	0.247	2.59	0.089	1.26	0.320	1.49	0.255	1.28	0.315
CS**	42.78	**0.001**	45.13	**0.001**	542.75	**0.001**	86.07	**0.001**	146.70	**0.001**	7.9	**0.013**
Y × CS	0.253	0.858	0.15	0.931	0.09	0.965	0.70	0.565	1.01	0.416	0.22	0.878

* All significant values ($p < 0.05$) are marked bold.
** Cropping systems include crops, e.g., cotton, maize, sugarcane, and soybean, under soybean-based intercropping systems (cotton/soybean, maize/soybean, and sugarcane/soybean) and their respective sole cropping systems.

Supplementary Table S13

Mixed model ANOVAs for the effects of years (Y) and cropping systems (CS) on land equivalent ratio for land ($pLER_L$), nitrogen ($pLER_N$), and phosphorus ($pLER_P$), and total land equivalent ratio for land (LER_L), nitrogen (LER_N), and phosphorus (LER_P) of soybean and main crops (cotton/maize/sugarcane).

Source	$pLER_N$				Total LER_N		$pLER_P$				Total LER_P		$pLER_L$				Total LER_L	
	Soybean		Main Crops				Soybean		Main Crops				Soybean		Main Crops			
	F	P	F	P	F	P	F	P	F	P	F	P	F	P	F	P	F	P
Y	1.23	0.322	0.20	0.894	0.76	0.527	0.74	0.541	4.57	**0.011***	2.58	0.077	1.66	0.202	0.60	0.622	2.54	0.080
CS**	21.67	**0.001**	15.74	**0.001**	51.80	**0.001**	135.19	**0.001**	119.12	**0.001**	317.45	**0.001**	15.99	**0.001**	30.94	**0.001**	47.10	**0.001**
Y × CS	0.166	0.983	0.128	0.992	0.16	0.986	0.64	0.700	3.13	**0.021**	1.80	0.141	0.28	0.939	0.21	0.969	0.54	0.771

* All significant values ($p < 0.05$) are marked bold.

** Cropping systems include three different soybean-based intercropping systems (cotton/soybean, maize/soybean, and sugarcane/soybean).

Supplementary Table S14

Mixed model ANOVAs for the effects of years (Y) and cropping systems (CS) on estimated nitrogen requirement (ENR), estimated phosphorus requirement (EPR), potential nitrogen reduction (PNR), potential phosphorus reduction (PPR), potential cost savings on nitrogen (PCS_N), potential cost savings on phosphorus (PCS_P), and potential total cost savings on nitrogen and phosphorus fertilizers ($PTCS_{NP}$).

Source	Estimated N and P requirement				Potential N and P reductions				Potential cost savings on N and P fertilizers				$PTCS_{NP}$	
	ENR		EPR		PNR		PPR		PCS_N		PCS_P			
	F	P	F	P	F	P	F	P	F	P	F	P	F	P
Y	0.74	0.537	2.80	0.061	0.75	0.535	2.55	0.080	48.53	**0.001***	35.16	**0.001**	95.56	**0.001**
CS**	58.84	**0.001**	21.52	**0.001**	58.86	**0.001**	785.75	**0.001**	52.40	**0.001**	702.11	**0.001**	544.82	**0.001**
Y × CS	0.13	0.992	2.03	0.101	0.13	0.992	1.87	0.127	0.93	0.493	4.55	**0.003**	3.99	**0.007**

* All significant values ($p < 0.05$) are marked bold.

** Cropping systems include three different soybean-based intercropping systems (cotton/soybean, maize/soybean, and sugarcane/soybean).

Supplementary Table S15
Value-based indices for soybean-based intercropping systems (4-year averages)

Intercropping system	System gross value (USD ha^{-1})*	Monetary advantage index (USD ha^{-1})**
Cotton/soybean	2359	+807
Maize/soybean	2430	+603
Sugarcane/soybean	4406	+1829

* & ** These indices, calculated from 4-year average yields (Tables 1-2), LER values (Table 4), and annual market prices (Table 6)
